# P-1241. Cefepime Pharmacodynamics Against *Pseudomonas aeruginosa* Evaluated in a Chemostat Infection Model: Do Generalized Cephalosporin Targets Translate?

**DOI:** 10.1093/ofid/ofae631.1423

**Published:** 2025-01-29

**Authors:** Mitchell Edwards, Daniel C Pevear, Fan Yi, Denis M Daigle, Lindsay M Avery

**Affiliations:** Venatorx Pharmaceuticals, Malvern, Pennsylvania; Venatorx Pharmaceuticals, Malvern, Pennsylvania; Venatorx Pharmaceuticals, Malvern, Pennsylvania; Venatorx Pharmaceuticals Inc., Street, Maryland; Venatorx Pharmaceuticals, Malvern, Pennsylvania

## Abstract

**Background:**

Cefepime (FEP) is used widely to treat infections caused by susceptible strains of *Pseudomonas aeruginosa*. However, nonclinical pharmacokinetic-pharmacodynamic (PK/PD) relationships for this drug-bug combination are not fully characterized. An in vitro chemostat (CS) model was used to assess the translatability of broadly established cephalosporin PK/PD targets to support development of FEP combined with taniborbactam (TAN), a novel serine- and metallo-β-lactamase inhibitor.

**Table.**
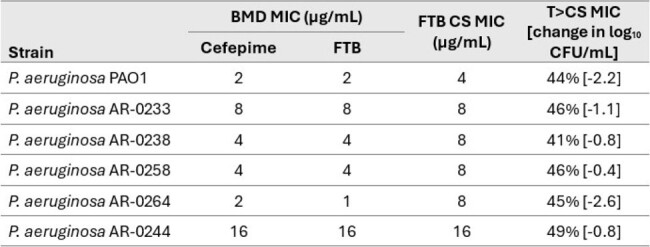
Antimicrobial susceptibility testing results and summary of cefepime pharmacodynamics against strains evaluated in the chemostat (CS) model AR, denotes strains sourced from the CDC & FDA Antimicrobial Resistance Isolate Bank; FTB, cefepime-taniborbactam

**Methods:**

Cefepime-taniborbactam (FTB) and FEP minimum inhibitory concentrations (MIC) were determined by reference broth microdilution (BMD) against *P. aeruginosa* isolates with no known acquired β-lactamases (N=6). Cefepime MICs were also assessed in 125 mL of TAN-supplemented media (4 µg/mL) to simulate CS conditions (CS MIC, Figure). In FEP dose-ranging studies in the dynamic CS model, bioreactors were inoculated with ≥ 5 log_10_ CFU/mL. FEP was dosed every 8 h for 24 h while media was infused to achieve a 2-h half-life. FEP concentrations were determined by a qualified bioanalytical method and used to calculate the percentage of time concentrations exceeded MIC values (T > MIC). Viable bacteria were quantified by serial dilution and plating at time points from 0 to 24 h. CS qualification was performed with Enterobacterales to confirm that the model recapitulated in vivo PD profiles reported previously by others.

**Figure.**
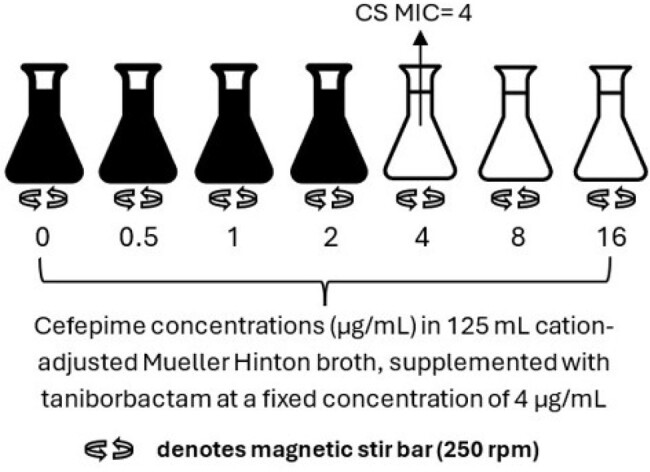
Schematic of the chemostat MIC (CS MIC) assay for an example strain with a CS MIC of 4 µg/mL.

**Results:**

*P. aeruginosa* strains were FEP-susceptible (n=5) and -nonsusceptible (n=1) and BMD MICs ranged from 2 to 16 µg/mL. CS MICs were equal to (n=2), 2x (n=3), or 8x (n=1) the FTB BMD MIC (Table). All strains grew in the CS model to an average (standard deviation) bacterial burden of 7.3 (0.6) log_10_ CFU/mL. In composite PD models fit to dose-ranging data, change in bacterial burden from 0 to 24 h was best described by T > CS MIC versus T > BMD MIC. Reductions in bioburden were observed at T > CS MICs of 41-49% (Table).

**Conclusion:**

In a dynamic in vitro model of *P. aeruginosa* infection, efficacious FEP exposures were similar to those established for Enterobacterales when indexed to CS MIC values. The results also indicate that differences between BMD and CS MIC should be considered when performing in vitro PK/PD profiling of discovery-phase antibacterials to prevent underestimation of therapeutic potential.

**Disclosures:**

**All Authors**: No reported disclosures

